# Development a risk prediction nomogram for multidrug−resistant bacterial and fungal infection in gastrointestinal fistula patients during the perioperative period

**DOI:** 10.3389/fcimb.2024.1502529

**Published:** 2024-11-28

**Authors:** Mingming Yin, Haoyi Zheng, Lifeng Xu, Rong Jin, Xiangyang Wang, Yi Man, Kai Xu, Qiang Ruan, Ting Wang, Kai Guo, Zheng Zhou, Wenyong Wu, Guosheng Gu

**Affiliations:** ^1^ Department of General Surgery, Anhui No.2 Provincial People’s Hospital, Hefei, China; ^2^ Department of General Surgery, The Graduate School of Bengbu Medical University, Bengbu, China

**Keywords:** multidrug-resistant infections, gastrointestinal fistulas, gram-negative bacilli, nomogram, perioperative period

## Abstract

**Background:**

This study aims to develop a risk prediction model for multidrug-resistant bacterial and fungal infections in patients with gastrointestinal fistulas during the perioperative period.

**Methods:**

A retrospective cohort study was conducted at Anhui No. 2 Provincial People’s Hospital from January 2022 to July 2024. We analyzed the distribution, resistance patterns, and mechanisms of multidrug resistance. Univariate and multivariate logistic regression analyses were performed to identify independent risk factors. A nomogram was constructed based on these risk factors, and its performance was evaluated using calibration curves, receiver operating characteristic (ROC) curves, and decision curve analysis (DCA).

**Results:**

A total of 266 patients were included, with 157 (59.02%) testing positive for multidrug-resistant infections. We isolated 329 pathogenic strains: 84 Gram-positive (25.53%), 215 Gram-negative (65.35%), and 30 fungal strains (9.11%). The most common isolate was *Klebsiella pneumoniae* (57 strains, 17.33%). Patients were divided into a training cohort (n = 177) and a validation cohort (n = 89). Multivariate analysis identified six key indicators: secondary surgery, length of hospital stay, preoperative white blood cell (WBC) count, preoperative neutrophil count, postoperative WBC count, and postoperative C-reactive protein (CRP) levels. The nomogram demonstrated excellent predictive ability, with an area under the curve (AUC) of 0.905 in the training cohort and 0.793 in the validation cohort. Calibration curves indicated high consistency between predicted probabilities and observed values. DCA confirmed the clinical utility of the nomogram.

**Conclusion:**

Our study shows that multidrug-resistant infections in patients with gastrointestinal fistulas are predominantly caused by Gram-negative bacilli, especially carbapenem-resistant Enterobacteriaceae. Key risk factors include secondary surgery and various blood count parameters. The developed nomogram provides robust predictive accuracy, aiding healthcare providers in implementing targeted infection prevention strategies.

## 1 Introduction

Gastrointestinal fistula is an abnormal connection between the gastrointestinal tract and the abdominal cavity, body surface, or other organs. It can be primarily classified into types such as gastrointestinal fistula, enteric-cutaneous fistulas, enteric-internal fistulas, and entero-atmospheric fistulas (EAF) (Gribovskaja-Rupp and Melton, 2016; Lee and Loi, 2024). Surgical procedures are the main cause of gastrointestinal fistulas; however, other significant triggers include radiation therapy, inflammatory bowel disease, malignant tumors, and certain medications (Chiarello et al., 2022a, b; Zheng et al., 2020), The dangers of gastrointestinal fistulas primarily stem from the leakage of digestive fluids. On one hand, this can lead to severe electrolyte and acid-base imbalances; on the other hand, the digestive enzymes in these fluids can erode the surrounding tissue, potentially causing massive hemorrhage either in the abdominal cavity or the gastrointestinal tract. Furthermore, the leaked digestive fluids contain bacteria that may lead to infections in affected organs or regions, such as intra-abdominal infections, retroperitoneal infections, necrotizing fasciitis of the abdominal wall, urinary tract infections due to enterovesical fistulas, and reproductive system infections resulting from enterovaginal fistulas. Thus, gastrointestinal fistulas remain a challenging issue in clinical practice. A systematic review that included 3,078 patients with gastrointestinal fistulas revealed a high healing rate of 89%; however, the mortality rate reached 8.5%. Notably, gastrointestinal fistulas accompanied by severe intra-abdominal infections had a mortality rate close to 30% (Gefen et al., 2022). As research progresses, it has become increasingly clear that intra-abdominal infections associated with gastrointestinal fistulas are often the starting point for multiple high-risk factors leading to death. Anastomotic leaks are the most common cause of postoperative intra-abdominal infections, usually resulting from mixed infections by various microorganisms, including Enterococcus species, Enterobacteriaceae, Bacteroides, and fungi. Additionally, the prolonged use of broad-spectrum antibiotics has resulted in many bacteria exhibiting multidrug resistance, complicating clinical management of gastrointestinal fistula patients (Mücke et al., 2017; Liu et al., 2017; Wu et al., 2015). Current guidelines recommend empirical antibiotic treatment strategies that only partially cover resistant microorganisms, making infections caused by multidrug-resistant bacteria often complex and challenging to treat. Consequently, clinicians face difficulties in selecting effective antimicrobial treatment strategies (Roson-Calero et al., 2023).

The treatment methods for gastrointestinal fistulas have been progressively improved and refined through clinical exploration. Staged treatment strategies have been validated in clinical practice, benefiting patients with gastrointestinal fistulas. The overall treatment of gastrointestinal fistulas can be broadly divided into three phases: the early phase of infection control and resuscitation, the non-surgical treatment phase, and the elective definitive surgery phase. Significant advances in technologies such as vacuum-assisted closure (VAC), over the scope clip (OTSC), dual-lumen drainage devices, and enteral and parenteral nutrition have supported non-surgical management. However, many fistulas continue to persist without healing spontaneously. When conditions such as the release of abdominal adhesions, improved nutritional status, and restoration of organ function are met, definitive surgery can be implemented. This underscores the importance of surgical intervention as a critical approach in the treatment of gastrointestinal fistulas (Jeong et al., 2023).

Therefore, it is crucial to investigate the prevalence of multidrug-resistant (MDR) infections in the perioperative period of patients with gastrointestinal fistulas. However, there is currently a lack of relevant data for this population. Through a retrospective study, we assessed the specifics of MDR bacterial and fungal infections, their resistance patterns, and conducted a thorough analysis of associated risk factors. We aimed to effectively integrate this information to establish a risk prediction nomogram, providing a foundation and reference for the diagnosis and treatment of MDR bacterial and fungal infections in patients with gastrointestinal fistulas during the perioperative period.

## 2 Patients and methods

### 2.1 Patients

From January 2022 to July 2024, a total of 266 patients with gastrointestinal fistulas were enrolled at the Anhui No. 2 Provincial People’s Hospital. The inclusion criteria were as follows: (1) Diagnosis of gastrointestinal fistula: any discharge from the gastrointestinal tract, which may include abdominal abscesses or spontaneous drainage through a stoma (Gefen et al., 2022); (2) Undergoing surgical treatment during hospitalization; (3) Perioperative cultures of sputum, blood, pus, bile, etc., indicating infections with multidrug-resistant bacteria and fungi; (4) Diagnosis of multidrug-resistant bacteria: bacteria that exhibit resistance to three or more commonly used antimicrobial agents that are typically sensitive (Rafailidis and Kofteridis, 2021). The exclusion criteria were: (1) Patients with gastrointestinal fistulas who did not receive surgical treatment; (2) Patients who died within 24 hours post-surgery or were discharged. The included cases were randomly divided into two groups in a 2:1 ratio, creating a training cohort and a validation cohort. The training cohort was used for developing the predictive model, while the validation cohort was used to assess its predictive performance. This study protocol adheres to the ethical standards of the Declaration of Helsinki and has been approved by the ethics committee of the China Clinical Trial Registration Center. In accordance with the recommendations of the ethics committee of the Anhui No. 2 Provincial People’s Hospital, written informed consent was obtained from all participants.

### 2.2 Data collection

Patient data were collected through the hospital information system, including gender, age, specific category of gastrointestinal fistula, date of surgery, duration of surgery, type of surgery (emergency/elective), surgical level (Grade III/IV), length of hospital stay, ICU admission duration, presence of reoperation, and comorbidities such as diabetes, hypertension, and malignancy. Clinical outcomes, including mortality, were also recorded. Data on multidrug-resistant organisms included specimen types, bacterial names, antibiotic susceptibility testing, and report dates. Preoperative clinical data comprised white blood cell counts, neutrophil counts, hemoglobin levels, platelet counts, albumin levels, C-reactive protein levels, total bilirubin, alanine aminotransferase, creatinine, procalcitonin, D-dimer levels, as well as postoperative laboratory values for white blood cells, neutrophils, hemoglobin, platelets, albumin, C-reactive protein, total bilirubin, alanine aminotransferase, creatinine, procalcitonin, and D-dimer. We defined “postoperative” as the most recent test result for specimens indicating multidrug-resistant bacteria or fungal infections. If there was no such infection, we considered relevant test results from 5 to 10 days post-surgery.

### 2.3 Statistical analysis

The raw data were analyzed using R software (version 3.6.1). For normally distributed continuous variables, the results are presented as means ± standard deviations (mean, ± SD). Categorical data are expressed as counts, rates, and percentages, while ordinal data are reported as counts and percentages. Univariate logistic regression analysis and multivariate logistic regression analysis were employed to identify independent influencing factors. Based on the results of the logistic regression analysis, a nomogram was created using the *regplot* package. The *pROC* package was utilized to generate the ROC curve, with the area under the curve (AUC) used to evaluate the discriminative ability of the nomogram model. Calibration of models was measured by Hosmer−Lemeshow goodness−of−fit test and calibration plot. Decision curve analysis (DCA) was performed using the *rmda* package to evaluate the clinical applicability of the nomogram model. A p-value of <0.05 was considered statistically significant.

## 3 Results

### 3.1 Analysis of multidrug-resistant bacteria and fungi

This study included a total of 266 patients with gastrointestinal fistula, all of whom underwent surgical treatment. Among these patients, 157 (59.02%) tested positive for infections caused by multidrug-resistant bacteria and fungi, with an average time to positivity of 6.36 ± 13.06 days. In contrast, 109 (40.98%) patients tested negative for such infections. A total of 329 pathogenic strains were isolated, comprising 84 Gram-positive strains (25.53%), 215 Gram-negative strains (65.35%), and 30 fungal strains (9.11%).

**Figure 1:**
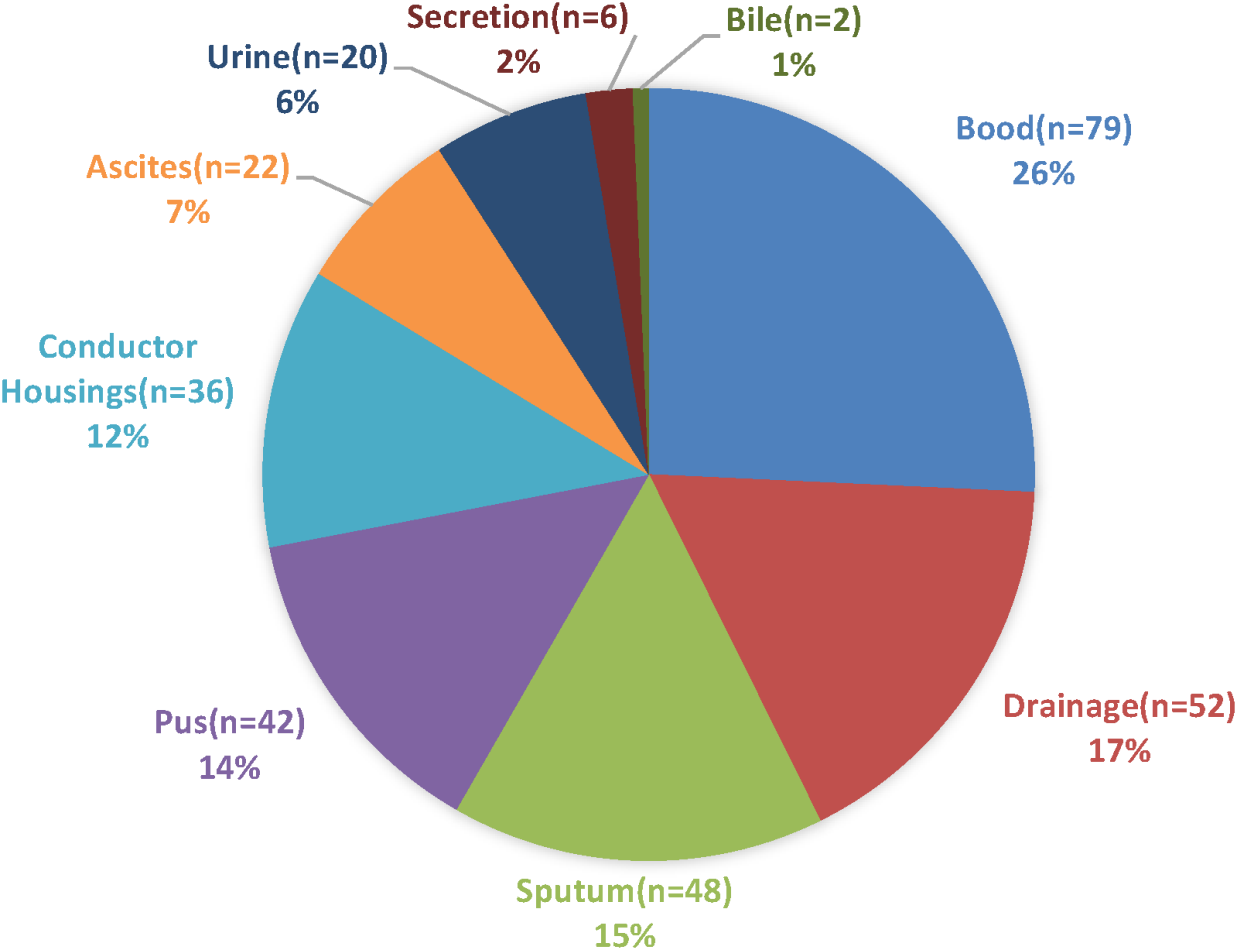
Distribution of strain groups identified in the study.

**Figure 2:**
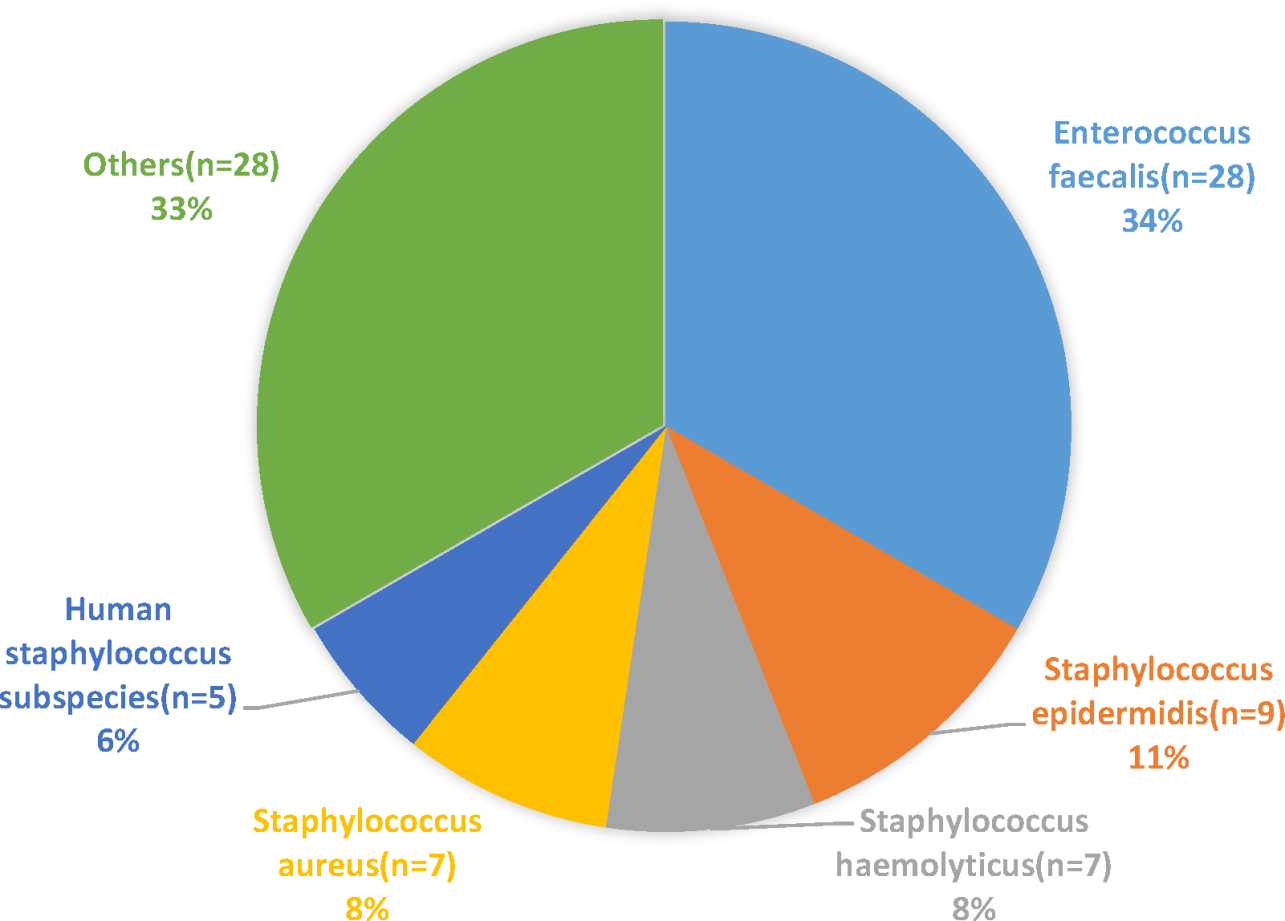
Distribution of Gram-positive bacteria identified in the study.

**Figure 3:**
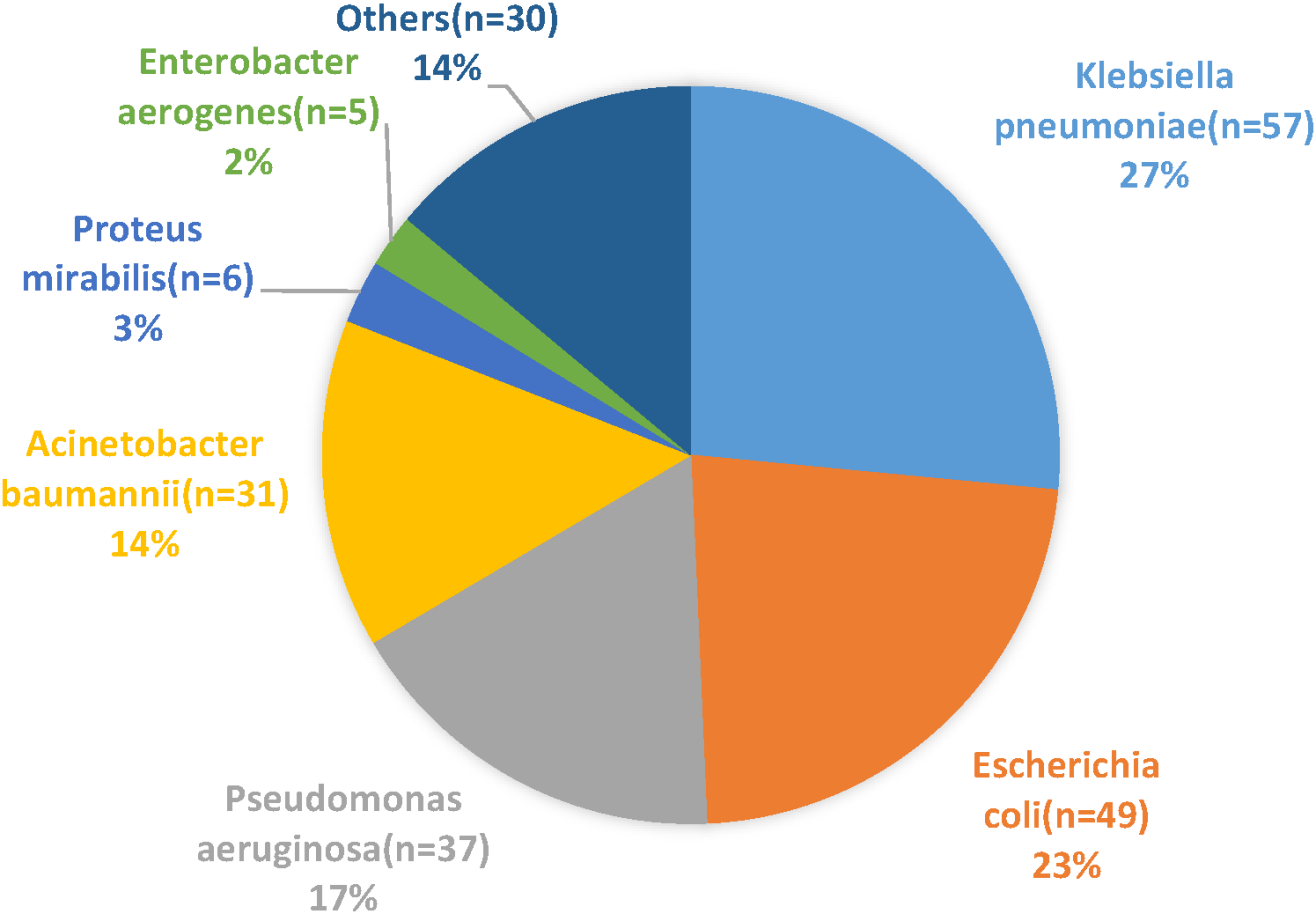
Distribution of Gram-negative bacteria identified in the study.

**Figure 4:**
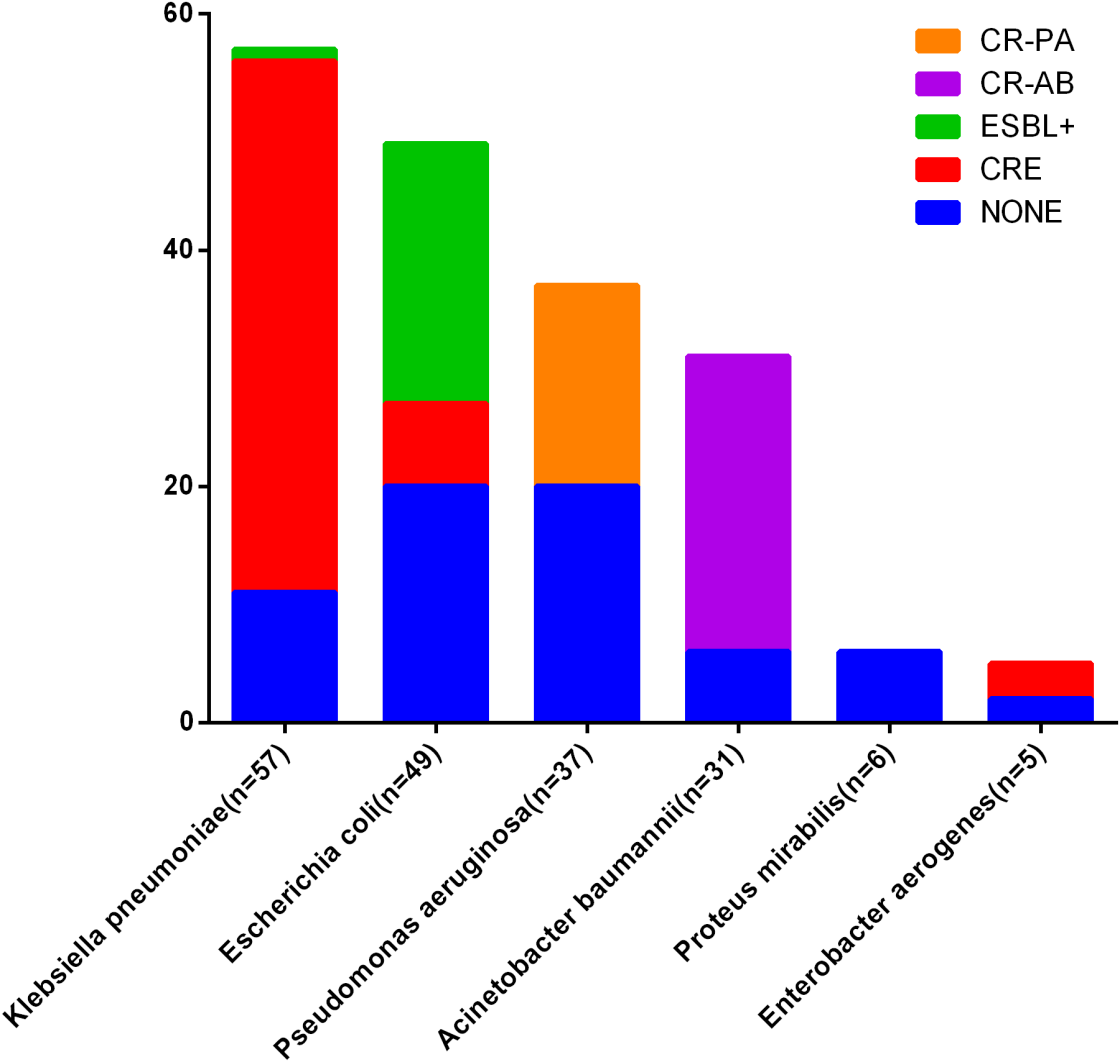
Antimicrobial resistance mechanisms in major Gram-negative bacteria. CRE, Carbapenem-resistant Enterobacteriaceae, CR-PA, Carbapenem-resistant Pseudomonas aeruginosa; CR-AB, Carbapenem-resistant Acinetobacter baumannii; ESBL+, Extended-Spectrum Beta-lactamases+.

Overall, *Klebsiella pneumoniae* was the most frequently isolated bacterial microorganism, accounting for 57 strains (17.33% of all bacterial isolates) and 26.51% of Gram-negative isolates. This was followed by *Escherichia coli*, which accounted for 49 strains (14.89% of all bacterial isolates) and 22.79% of Gram-negative isolates, as detailed in **Table 1**. The sources of the multidrug-resistant bacterial and fungal strains included blood (26%), drainage (17%), sputum (15%), pus (14%), and conductor housings (12%), as detailed in **Figure 1**. The distribution of Gram-positive and Gram-negative bacteria is illustrated in **Figures 2**, **3**. Statistical analyses were conducted to evaluate the resistance patterns of the main multidrug-resistant bacteria, with results summarized in **Tables 2**, **3**. The distribution of resistance mechanisms for major Gram-negative bacteria is depicted in **Figure 4**.

**Table 1:** Constituent ratios of the isolated MDRB and fungus (%).

Pathogenic Bacteria	Number of Strains	Ratio (%)
Gram-positive bacteria		
*Enterococcus faecalis*	28	8.51%
*Staphylococcus epidermidis*	9	2.74%
*Staphylococcus haemolyticus*	7	2.13%
*Staphylococcus aureus*	7	2.13%
*Human staphylococcus subspecies*	5	1.52%
*Enterococcus raffinosus*	4	1.22%
*Staphylococcus sciuri*	4	1.22%
*Staphylococcus warneri*	4	1.22%
*Enterococcus gallinarum*	3	0.91%
*Staphylococcus capitis*	3	0.91%
*Enterococcus vulgare*	3	0.91%
*Streptococcus anginosus*	2	0.61%
*Others*	5	1.52%
Gram-negative bacteria		
*Klebsiella pneumoniae*	57	17.33%
*Escherichia coli*	49	14.89%
*Pseudomonas aeruginosa*	37	11.25%
*Acinetobacter baumannii*	31	9.42%
*Proteus mirabilis*	6	1.82%
*Enterobacter aerogenes*	5	1.52%
*Stenotrophomonas maltophilia*	4	1.22%
*Enterobacter cloacae*	3	0.91%
*Serratia marcescens*	3	0.91%
*Klebsiella oxytoca*	2	0.61%
*Citrobacter freundii*	2	0.61%
*Corynebacterium striatum*	2	0.61%
*pseudomonas fluorescens*	2	0.61%
*Others*	12	3.65%
Fungus		
*Candida albicans*	9	2.74%
*Candida parapsilosis*	7	2.13%
*Candida glabrata*	6	1.82%
*Candida tropicalis*	6	1.82%
*Candida guilliermondii*	1	0.30%
*Filamentous fungi*	1	0.30%
Total	329	100%

**Table 2:** Antimicrobial resistance of Gram-positive bacteria isolates to the tested antibiotics.

Antibacterial agents	*Enterococcus faecalis* (n=28)		*Staphylococcus epidermidis* (n=9)		*Staphylococcus haemolyticus* (n=7)		*Staphylococcus aureus* (n=7)		*Human staphylococcus subspecies* (n=5)	
	Number	Resistance rate (%)	Number	Resistance rate (%)	Number	Resistance rate (%)	Number	Resistance rate (%)	Number	Resistance rate (%)
gentamicin	19	67.86%	1	11.11%	5	71.43%	3	42.86%	0	0.00%
levofloxacin	25	89.29%	4	44.44%	6	85.71%	4	57.14%	4	80.00%
tigacycline	1	3.57%	0	0.00%	1	14.29%	0	0.00%	0	0.00%
ciprofloxacin	3	10.71%	null	null	3	42.86%	null	null	0	0.00%
datomycin	0	0.00%	0	0.00%	0	0.00%	0	0.00%	0	0.00%
erythromycin	21	75.00%	7	77.78%	6	85.71%	5	71.43%	4	80.00%
linezolid	0	0.00%	0	0.00%	0	0.00%	0	0.00%	0	0.00%
penicillin	23	82.14%	9	100.00%	7	100.00%	6	85.71%	5	100.00%
rifampicin	3	10.71%	0	0.00%	3	42.86%	1	14.29%	0	0.00%
tetracycline	4	14.29%	null	null	2	28.57%	null	null	0	0.00%
vancomycin	0	0.00%	0	0.00%	0	0.00%	0	0.00%	0	0.00%
average antibiotic resistance	99	32.14%	21	23.33%	33	42.86%	19	30.16%	13	23.64%

**Table 3:** Antimicrobial resistance of Gram- negative bacteria isolates to the tested antibiotics.

Antibacterial agents	*Klebsiella pneumoniae* (n=57)		*Escherichia coli* (n=49)		*Pseudomonas aeruginosa* (n=37)		*Acinetobacter baumannii* (n=31)		*Proteus mirabilis* (n=6)		*Enterobacter aerogenes* (n=5)	
	Number	Resistance rate (%)	Number	Resistance rate (%)	Number	Resistance rate (%)	Number	Resistance rate (%)	Number	Resistance rate (%)	Number	Resistance rate (%)
amoxicillin	33	57.89%	7	14.29%	1	2.70%	null	null	0	0.00%	1	20.00%
piperacillin/Tazobactam	49	85.96%	15	30.61%	6	16.22%	28	0.903226	1	16.67%	4	80.00%
piperacillin	12	21.05%	18	36.73%	3	8.11%	3	0.096774	2	33.33%	0	0.00%
ampicillin/sulbactam	28	49.12%	17	34.69%	4	10.81%	28	0.903226	1	16.67%	null	null
ampicillin	10	17.54%	37	75.51%	4	10.81%	2	0.064516	4	66.67%	null	null
cefazolin	44	77.19%	40	81.63%	5	13.51%	3	0.096774	3	50.00%	null	null
cefuroxime	44	77.19%	42	85.71%	4	10.81%	3	9.68%	4	66.67%	2	40.00%
cefoxitin	3	5.26%	10	20.41%	null	null	null	null	1	16.67%	null	null
cefoperazone and sulbactam	47	82.46%	16	32.65%	10	27.03%	24	77.42%	0	0.00%	4	80.00%
ceftazidime	52	91.23%	32	65.31%	10	27.03%	29	93.55%	2	33.33%	4	80.00%
cefepime	51	89.47%	40	81.63%	3	8.11%	24	77.42%	2	33.33%	3	60.00%
ceftriaxone	11	19.30%	22	44.90%	null	null	null	null	3	50.00%	null	null
cefotaxime	14	24.56%	23	46.94%	4	10.81%	6	19.35%	1	16.67%	0	0.00%
aztreonam	49	85.96%	39	79.59%	10	27.03%	20	64.52%	0	0.00%	2	40.00%
imipenem	48	84.21%	7	14.29%	20	54.05%	28	90.32%	1	16.67%	4	80.00%
meropenem	47	82.46%	4	8.16%	14	37.84%	27	87.10%	0	0.00%	4	80.00%
amikacin	26	45.61%	0	0.00%	1	2.70%	23	74.19%	0	0.00%	3	60.00%
gentamicin	11	19.30%	12	24.49%	2	5.41%	7	22.58%	1	16.67%	2	40.00%
levofloxacin	50	87.72%	35	71.43%	9	24.32%	25	80.65%	3	50.00%	3	60.00%
tigacycline	1	1.75%	0	0.00%	28	75.68%	2	6.45%	6	100.00%	1	20.00%
doxycycline	16	28.07%	11	22.45%	16	43.24%	13	41.94%	null	null	1	20.00%
trimethoprim	20	35.09%	24	48.98%	22	59.46%	16	51.61%	5	83.33%	0	0.00%
polymyxin B	3	5.26%	0	0.00%	0	0.00%	0	0.00%	null	null	0	0.00%
ceftazidime and avibactam	6	10.53%	2	4.08%	1	2.70%	null	null	null	null	0	0.00%
average antibiotic resistance	675	49.34%	453	38.52%	177	21.74%	311	50.16%	40	31.75%	38	40.00%

### 3.2 Patient characteristics

A total of 266 hospitalized patients who met the inclusion criteria were enrolled in the study and randomly divided into a training cohort (n = 177) and a validation cohort (n = 89) at a ratio of 2:1. The clinical and pathological characteristics of the training and validation Cohort are compared in **Table 4**. Aside from postoperative procalcitonin (PCT) and postoperative creatinine (Cr) levels, there were no significant differences in other indicators between the two groups (**Table 4**).

**Table 4:** Characteristics of patients in the training and validation cohorts.

	Training cohort (n=177)	Validation cohort (n=89)	P
Age, years (mean, ± SD)	56.23 ± 16.02	55.22 ± 15.77	0.629
Gender, n (%)			
Female	79	41	
Male	98	48	0.896
Duration of surgery (mean, ± SD)	194.22 ± 84.51	195.29 ± 75.73	0.92
Type of surgery			
Emergency operation	50	23	
Selective operation	127	66	0.678
Grade of surgery			
III	100	43	
IV	77	46	0.207
Secondary surgery			
Yes	18	15	
No	159	74	0.119
Categories of gastrointestinal fistula			
colon fistula	41	25	
enteric fistula	86	38	
colon-enteric fistula	9	10	
gastric fistula	3	5	
duodenal fistula	13	6	
others	25	14	0.247
With malignant tumors			
Yes	89	47	
No	88	42	0.697
With hypertension			
Yes	23	8	
No	154	81	0.337
With diabetes			
Yes	13	10	
No	164	79	0.287
Clinical outcome			
Survival	145	76	
Death	32	13	0.476
ICU stays (mean, ± SD)	6.19 ± 9.72	4.81 ± 5.70	0.219
Hospital stays (mean, ± SD)	50.42 ± 30.67	53.42 ± 42.87	0.514
Preoperative WBC (×10^9^/L)	7.83 ± 5.50	8.16 ± 4.89	0.163
Preoperative N (×10^9^/L)	6.01 ± 5.25	6.42 ± 4.88	0.104
Preoperative L (×10^9^/L)	1.61 ± 6.48	1.09 ± 0.61	0.653
Preoperative Hb (g/L)	101.85 ± 22.91	106.42 ± 21.93	0.130
Preoperative PLT (×10^9^/L)	211.50 ± 108.22	219.54 ± 93.79	0.421
Preoperative ALB (g/L)	34.25 ± 6.10	34.87 ± 5.94	0.413
Preoperative CRP (mg/L)	68.82 ± 85.08	60.44 ± 85.52	0.303
Preoperative TBIL (umol/L)	19.34 ± 21.64	19.27 ± 24.18	0.448
Preoperative ALT (U/L)	34.69 ± 35.55	35.83 ± 33.25	0.894
Preoperative Cr (umol/L)	75.24 ± 63.27	63.06 ± 36.39	0.183
Preoperative PCT (ng/L)	8.06 ± 28.27	2.12 ± 6.50	0.415
Preoperative D-dimer (mg/L)	2.92 ± 3.91	2.83 ± 5.53	0.574
Postoperative WBC (×10^9^/L)	11.10 ± 6.98	10.20 ± 5.68	0.581
Postoperative N (×10^9^/L)	10.25 ± 10.55	8.56 ± 5.42	0.649
Postoperative L (×10^9^/L)	1.02 ± 0.91	0.97 ± 0.61	0.797
Postoperative Hb (g/L)	93.45 ± 17.55	93.37 ± 16.67	0.071
Postoperative PLT (×10^9^/L)	213.38 ± 121.52	222.62 ± 113.49	0.601
Postoperative ALB (g/L)	33.07 ± 5.90	33.74 ± 5.89	0.288
Postoperative CRP (mg/L)	102.25 ± 99.67	89.61 ± 91.26	0.237
Postoperative TBIL (umol/L)	30.87 ± 41.99	25.36 ± 28.11	0.331
Postoperative ALT (U/L)	55.23 ± 137.38	38.34 ± 39.42	0.702
Postoperative Cr (umol/L)	75.73 ± 65.53	55.90 ± 32.09	0.003
Postoperative PCT (ng/L)	5.39 ± 13.42	1.36 ± 4.05	0.005
Postoperative D-dimer (mg/L)	4.25 ± 4.29	3.60 ± 3.39	0.846

The colored values indicate P-values less than 0.05, highlighting statistically significant results.

### 3.3 Risk factors associated with multidrug−resistant bacterial and fungal infections

In the training cohort, 37 clinical indicators were analyzed for their association with multidrug-resistant bacterial and fungal infections. The analysis revealed that several factors, including age, type of surgery (emergency vs. selective), whether a secondary surgery was performed, clinical outcome (survival vs. death), length of ICU stay, length of hospital stay, and various preoperative and postoperative laboratory values (such as WBC count, neutrophil count, lymphocyte count, hemoglobin, albumin, CRP, TBIL, procalcitonin, and D-dimer), were significantly associated with these infections (P ≤ 0.05) (**Table 5**). Following variable selection through a multivariable regression model, the best predictors identified for multidrug-resistant bacterial and fungal infections included whether a secondary surgery was performed, length of hospital stay, preoperative WBC count, preoperative neutrophil count, postoperative WBC count, and postoperative CRP levels (**Table 6**).

**Table 5:** Risk factors for multidrug−resistant bacterial infections in the training cohort.

	With multidrug− resistant bacteria and fungus (n=105)	Without multidrug− resistant bacteria and fungus (n=72)	P
Age, years (mean, ± SD)	58.75 ± 15.68	52.54 ± 15.90	0.010
Gender, n (%)			
Female	42	37	
Male	63	35	0.134
Duration of surgery (mean, ± SD)	203.75 ± 93.93	180.32 ± 66.70	0.124
Type of surgery			
Emergency operation	36	14	
Selective operation	69	58	0.031
Grade of surgery			
III	63	27	
IV	42	35	0.256
Secondary surgery			
Yes	18	0	
No	87	72	<0.001
Categories of gastrointestinal fistula			
colon fistula	24	17	
enteric fistula	50	36	
colon-enteric fistula	4	5	
gastric fistula	3	0	
duodenal fistula	12	1	
others	12	6	0.104
With malignant tumors			
Yes	55	34	
No	50	38	0.500
With hypertension			
Yes	12	11	
No	93	61	0.454
With diabetes			
Yes	10	3	
No	95	69	0.180
Clinical outcome			
Survival	30	2	
Death	75	70	<0.001
ICU stays (mean, ± SD)	8.55 ± 11.56	2.74 ± 4.28	<0.001
Hospital stays (mean, ± SD)	58.37 ± 35.82	38.83 ± 14.92	<0.001
Preoperative WBC (×10^9^/L)	8.72 ± 6.27	6.52 ± 3.80	0.005
Preoperative N (×10^9^/L)	7.09 ± 5.92	4.45 ± 3.56	<0.001
Preoperative L (×10^9^/L)	1.79 ± 8.41	1.36 ± 0.65	<0.001
Preoperative Hb (g/L)	98.19 ± 20.59	107.19 ± 25.12	<0.001
Preoperative PLT (×10^9^/L)	217.09 ± 119.18	203.36 ± 90.07	0.636
Preoperative ALB (g/L)	33.25 ± 5.81	35.70 ± 6.29	0.001
Preoperative CRP (mg/L)	82.96 ± 92.32	29.76 ± 41.51	<0.001
Preoperative TBIL (umol/L)	22.31 ± 21.70	15.00 ± 20.96	<0.001
Preoperative ALT (U/L)	36.47 ± 41.88	32.10 ± 23.50	0.927
Preoperative Cr (umol/L)	73.88 ± 47.93	77.24 ± 80.95	0.870
Preoperative PCT (ng/L)	6.05 ± 19.70	0.31 ± 0.41	<0.001
Preoperative D-dimer (mg/L)	3.64 ± 4.16	1.34 ± 2.85	<0.001
Postoperative WBC (×10^9^/L)	13.82 ± 7.63	7.14 ± 2.98	<0.001
Postoperative N (×10^9^/L)	12.84 ± 10.07	6.47 ± 10.17	<0.001
Postoperative L (×10^9^/L)	1.00 ± 1.09	1.06 ± 0.55	0.019
Postoperative Hb (g/L)	90.41 ± 19.04	97.88 ± 14.11	0.005
Postoperative PLT (×10^9^/L)	207.76 ± 132.72	221.58 ± 103.38	0.176
Postoperative ALB (g/L)	31.36 ± 6.24	35.55 ± 4.31	<0.001
Postoperative CRP (mg/L)	126.38 ± 108.72	58.73 ± 60.79	<0.001
Postoperative TBIL (umol/L)	36.94 ± 47.36	22.02 ± 30.85	<0.001
Postoperative ALT (U/L)	66.58 ± 174.50	38.67 ± 41.37	0.365
Postoperative Cr (umol/L)	82.83 ± 66.68	65.38 ± 62.84	0.027
Postoperative PCT (ng/L)	7.02 ± 15.10	0.30 ± 0.31	<0.001
Postoperative D-dimer (mg/L)	4.91 ± 5.04	3.07 ± 1.93	0.265

The colored values indicate P-values less than 0.05, highlighting statistically significant results.

**Table 6:** Multivariate logistic analyses of risk factors for multidrug−resistant bacterial infections in the training cohort.

Variables	β	S.E	Z	*P*	OR (95%CI)
Intercept	-3.822	0.952	-4.014	<.001	0.022 (0.003 ~ 0.141)
Secondary surgery	18.299	1159.105	0.016	0.987	88510929.166 (0.000 ~ Inf)
Hospital stays	0.028	0.013	2.150	0.032	1.029 (1.002 ~ 1.055)
Preoperative WBC (×10^9^/L)	-0.763	0.269	-2.841	0.004	0.466 (0.275 ~ 0.789)
Preoperative N (×10^9^/L)	0.818	0.278	2.943	0.003	2.266 (1.314 ~ 3.908)
Postoperative WBC (×10^9^/L)	0.301	0.062	4.879	<.001	1.351 (1.197 ~ 1.525)
Postoperative CRP (mg/L)	0.012	0.004	3.259	0.001	1.012 (1.005 ~ 1.019)

OR, Odds Ratio; CI, Confidence Interval.

### 3.4 Construction of the multidrug−resistant bacterial and fungal infections−predicting nomogram

**Figure 5:**
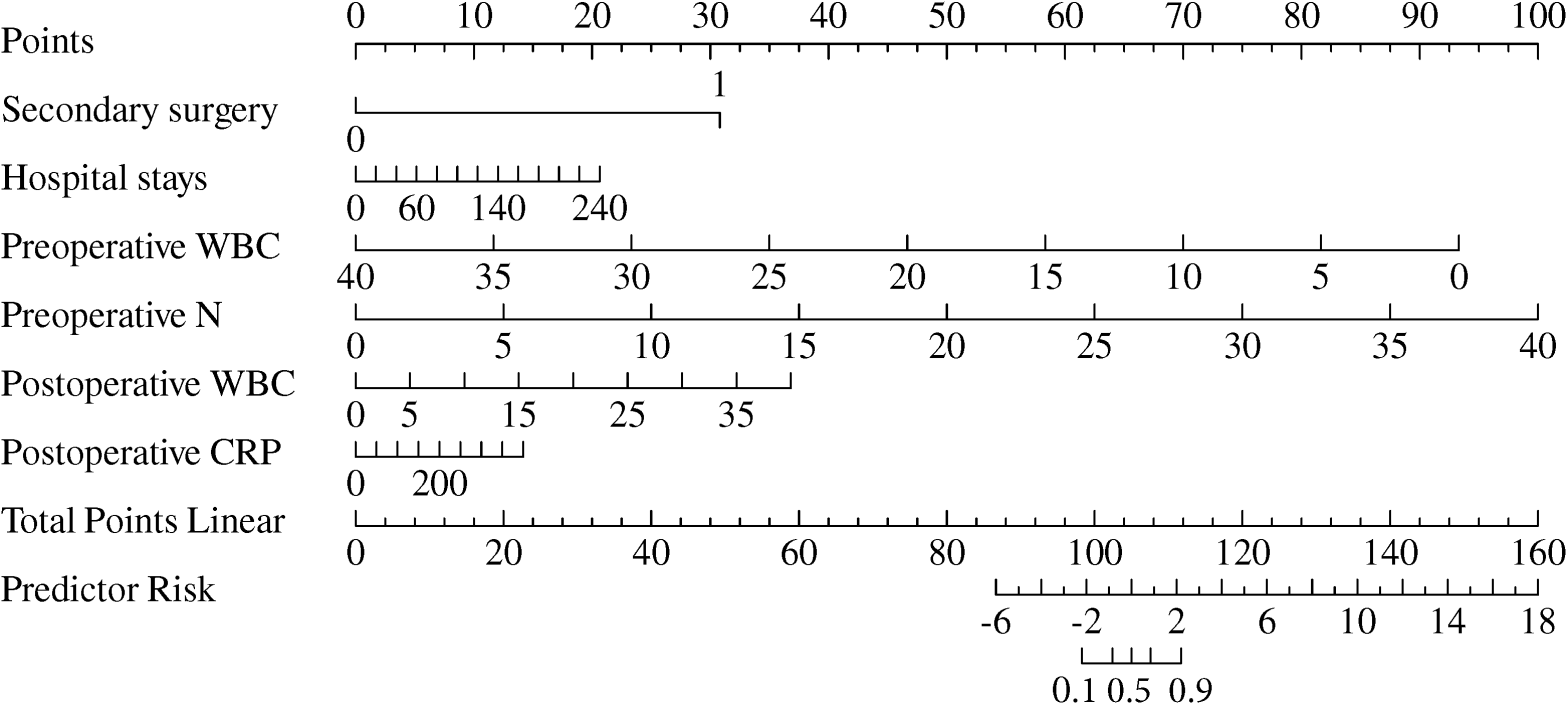
Nomogram predicting the probability of multidrug−resistant bacterial and fungal infections.

A nomogram was created to predict multidrug-resistant bacterial and fungal infections in patients with gastrointestinal fistulas, based on the independently identified risk factors from the multivariate logistic model (**Figure 5**). Each factor was assigned a specific weight in points. The total points for each patient were calculated using the nomogram, correlating with the estimated probability of multidrug-resistant bacterial infections. The preoperative neutrophil level received the highest score of 100 points, followed by preoperative WBC level (92.1 points), postoperative WBC count (34.2 points), secondary surgery (30.3 points), length of hospital stay (20.3 points), and postoperative CRP level (13.8 points).

### 3.5 Model performance assessment

**Figure 6:**
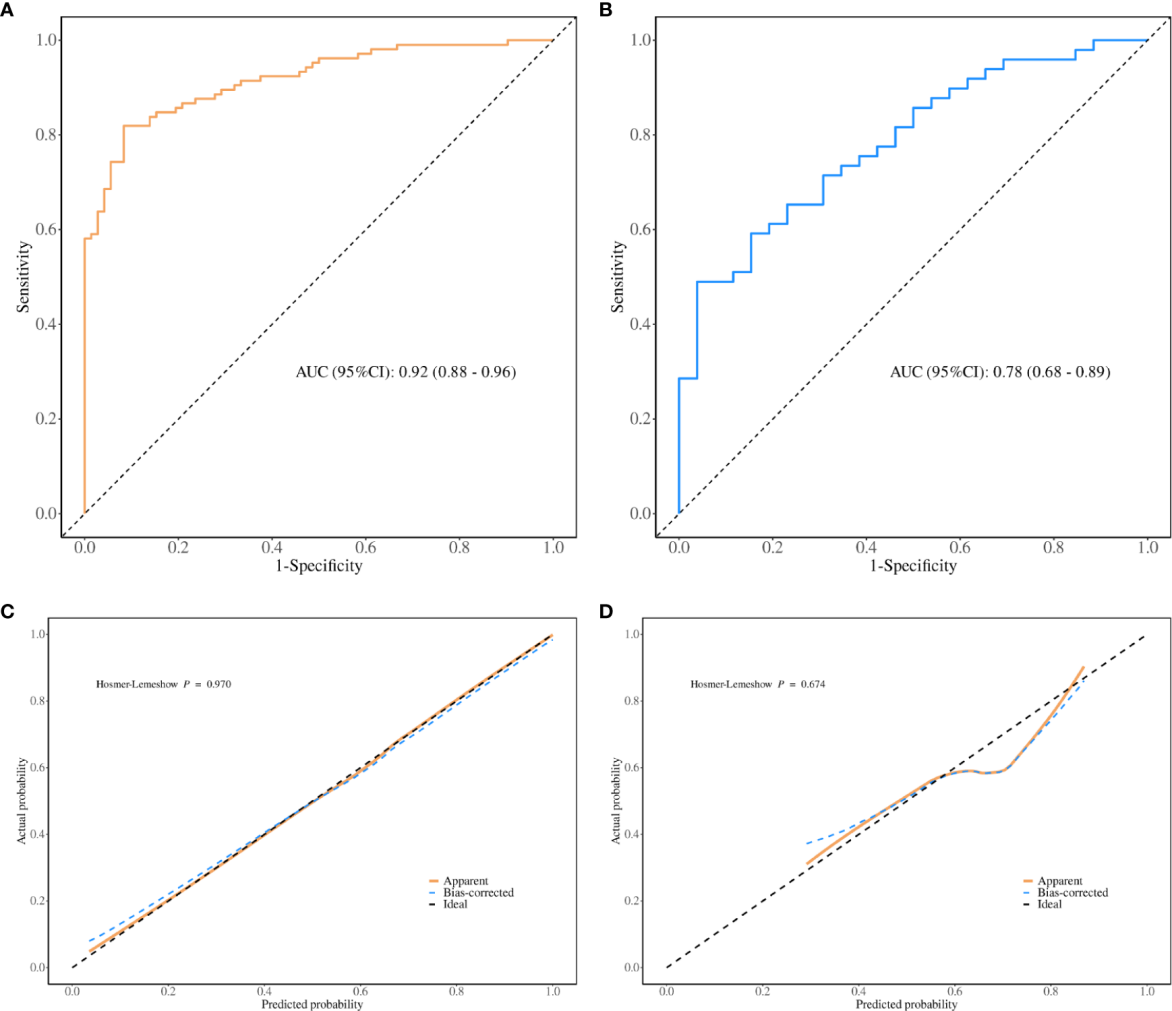
Goodness of Fit for Predicted and Actual Risks of Multidrug-Resistant and Fungal Infections. Panel **(A)** displays the ROC curves for the model in the training cohort, while panel **(B)** presents the ROC curves for the validation cohort. Panel **(C)** shows the calibration curves for the model in the training cohort, and panel **(D)** presents the corresponding calibration curves for the validation cohort. The ROC curves highlight the model’s discrimination capability, with a larger AUC indicating higher prediction accuracy. The calibration curves assess the alignment between the predicted risks of multidrug-resistant bacterial and fungal infections and the actual observed outcomes. The 45-degree dotted line represents perfect predictions, while the solid line indicates the model’s predictive performance. A closer fit of the solid line to the dotted line reflects better predictive accuracy.

**Figure 7:**
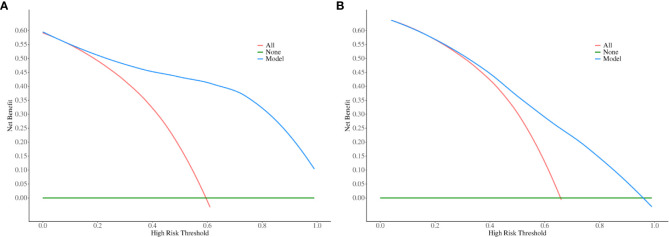
Decision curve analysis (DCA) for predicting multidrug-resistant bacterial and fungal infections. The x-axis represents the risk threshold probability, ranging from 0 to 1, while the y-axis shows the calculated net benefit associated with each threshold probability. The blue curves illustrate the net benefit of the model, whereas the red lines indicate the net benefits of strategies involving all patients with multidrug-resistant bacterial and fungal infections. In contrast, the green lines reflect the net benefits of strategies where no patients are assumed to have multidrug-resistant bacterial and fungal infections. Panels **(A, B)** correspond to the training cohort and the validation cohort, respectively.

The performance evaluation of the regression model is illustrated in **Figure 6**. The variance inflation factor (VIF) values for the seven covariates ranged from 1.203 to 2.079, indicating that there was no collinearity within the model. The area under the curve (AUC) was 0.92 (95% CI, 0.88–0.96), demonstrating excellent discrimination ability in the training cohort (**Figure 6A**). The calibration curve for the regression model based on the training cohort demonstrates strong performance, as confirmed by the Hosmer-Lemeshow test (P = 0.970) (**Figure 6C**). In this validation cohort, the AUC was 0.78 (95% CI, 0.68–0.89), indicating good model performance across both cohorts (**Figure 6B**). The calibration of the model for predicting multidrug-resistant bacterial infections was also validated in an independent cohort (**Figure 6D**), with the Hosmer-Lemeshow test yielding a non-significant P value of 0.674. The decision curve analysis (DCA) for the nomogram model is shown in **Figures 7A, B**. The nomogram demonstrated strong clinical applicability for predicted probabilities between 0.1 and 1.0. The accuracy rates were 82.5% in the training cohort and 69.3% in the validation cohort (**Table 7**).

**Table 7:** confusion matrix for training cohort and the validation cohort.

Data	AUC (95%CI)	Accuracy (95%CI)	Sensitivity (95%CI)	Specificity (95%CI)	PPV (95%CI)	NPV (95%CI)	Cut off
training cohort	0.905 (0.864-0.947)	0.825 (0.761-0.878)	0.889 (0.816 - 0.961)	0.781 (0.702 - 0.860)	0.736 (0.643 - 0.828)	0.911 (0.852 - 0.970)	0.624
validation cohort	0.793 (0.690-0.896)	0.693 (0.576-0.795)	0.692 (0.515 - 0.870)	0.694 (0.565 - 0.823)	0.545 (0.376 - 0.715)	0.810 (0.691 - 0.928)	0.624

## 4 Discussion

Gastrointestinal fistula is a common and severe complication of abdominal surgery. It represents a complex pathophysiological process associated with a range of serious complications and high mortality rates. In the 1970s, the mortality rate of gastrointestinal fistulas was approximately 50.0% to 60.0%. However, with ongoing research into the mechanisms of fistula formation and advancements in treatment methods, as well as improvements in medical technology, the current mortality rate has decreased to between 5.3% and 21.3% (Draus et al., 2006; Pepe et al., 2024). Studies have shown that patients with gastrointestinal fistulas often experience severe infections. This is primarily due to the disruption of intestinal integrity, which exposes a diverse array of microorganisms from the gut. Additionally, factors such as prolonged antibiotic use, extended hospital stays, long-term ICU admissions, and various invasive procedures significantly increase the risk of bacterial infections (Doerken et al., 2021). Bacterial infections are inextricably linked to the integrity of the intestinal mucosal barrier. However, enteral nutrition (EN) is superior to parenteral nutrition (PN) in maintaining the integrity of the intestinal mucosal barrier and regulating immune function (Grillo-Ardila et al., 2024; Shao et al., 2023). Patients with gastrointestinal fistulas, particularly those with high-output fistulas, often cannot receive EN early and must rely on PN management (Ortiz et al., 2017). During this period, the function of the intestinal mucosa is compromised to varying degrees, further heightening the risk of bacterial translocation and enteric infections (Casaer et al., 2011). Following severe infections, patients may exhibit related symptoms that adversely affect liver and kidney function, exacerbate malnutrition, and potentially lead to septic shock, creating a vicious cycle. In recent years, the application of numerous new technologies has enabled a significant number of patients suffering from gastrointestinal fistulas to improve with conservative treatment (Rabel et al., 2023). Nonetheless, surgical intervention remains critically important in the management of fistulas. Some patients can benefit from conservative treatment to improve their clinical and nutritional status, but complete fistula relief usually requires surgery (Huang et al., 2022). Furthermore, in the case of a duodenal or high jejunal fistula, aggressive surgical intervention is essential to prevent the exacerbation of infection or the risk of uncontrollable intra-abdominal hemorrhage caused by the corrosive output of the fistula, which can pose a serious threat to the patient’s life. This study focuses for the first time on the analysis of multidrug-resistant bacteria and fungi in patients with gastrointestinal fistulas during the perioperative period and aims to develop a risk prediction nomogram that assists clinicians in effectively assessing the risk of multidrug-resistant infections in patients requiring surgery. Treatment of infections caused by multidrug-resistant microorganisms can be enhanced by predicting infection by potential pathogens and selecting antibiotics based on drug sensitivity results.

In this study, we found that, the predominant pathogens associated with gastrointestinal fistula-related multi-drug resistant bacteria and fungal infections were Gram-negative bacilli, consistent with previous research (Liu et al., 2017). Among the Gram-negative bacteria, the leading strains identified were *Klebsiella pneumoniae, Acinetobacter baumannii*, and *Escherichia coli*. Notably, *Klebsiella pneumoniae* has now surpassed *Escherichia coli* to become the most prevalent strain, exhibiting the highest proportion of resistance mechanisms, including positivity for CRE (Carbapenem-resistant Enterobacteriaceae) and ESBL (Extended-Spectrum Beta-lactamases) at 80.7%. This trend necessitates significant clinical attention. The second most common strain was *Escherichia coli*, a typical member of the intestinal flora; however, a concerning proportion of these infections exhibited CRE or ESBL resistance mechanisms, reaching 59.2%. Additionally, the prevalence of CR-PA (Carbapenem-resistant Pseudomonas aeruginosa) and CR-AB (Carbapenem-resistant Acinetobacter baumannii) was notably high, at 45.9% and 80.6%, respectively. In recent years, carbapenem resistance has been linked to high mortality rates and has emerged as a global concern, increasing the treatment burden for infection control (Giacobbe et al., 2023; Boattini et al., 2023). Antimicrobial susceptibility testing revealed that the average antibiotic resistance rates for Klebsiella pneumoniae, Acinetobacter baumannii, and Escherichia coli were 49.34%, 50.16%, and 38.52%, respectively. The rates of resistance to meropenem among carbapenemase-producing Enterobacteriaceae were alarmingly high, with *Klebsiella pneumoniae, Acinetobacter baumannii*, and *Escherichia coli* showing resistance rates of 82.5%, 87.1%, and 81.6%, respectively, significantly exceeding previous reports (Sid Ahmed et al., 2023; Chang et al., 2023). Furthermore, we observed a concerning proportion of resistant strains to tigecycline, polymyxin B, and ceftazidime-avibactam in *Klebsiella pneumoniae*. This may be attributed to the fact that most of our patients were transferred from other hospitals and had already received several days of antibiotic treatment, which is a known risk factor for the development of carbapenem resistance (López-Cubillos et al., 2023). In terms of Gram-positive bacteria, the most common strain identified was *Enterococcus faecium*, accounting for 34%. We also discovered one case of methicillin-resistant *Staphylococcus aureus* (MRSA), two cases of methicillin-resistant coagulase-negative *staphylococci* (MRSCON), and one case of beta-lactamase-producing *Staphylococcus*. Among the cocci, the average resistance rates of *Enterococcus faecium* and *hemolytic Staphylococcus* to the tested antibiotics exceeded 30%. The resistance rate to penicillin in Gram-positive cocci exceeded 85%, and the resistance rate to the broad-spectrum antibiotic levofloxacin was also notably high. Thus far, there have been no observed resistance cases against linezolid or vancomycin among Gram-positive cocci; however, careful monitoring for related complications is warranted during their use. Fungal infections were relatively infrequent, comprising about 10% of cases, and no drug-resistant fungi were identified. Nonetheless, fungal infections can be clinically challenging to detect, and delays in antifungal treatment, prolonged use of antifungal medications, and substantial side effects pose significant difficulties in managing fungal infections associated with gastrointestinal fistula. Overall, this study highlights that in the perioperative period, patients with gastrointestinal fistula predominantly present with multidrug-resistant Gram-negative bacteria, particularly carbapenem-resistant Enterobacteriaceae, with *Klebsiella pneumoniae* emerging as a critical concern that requires heightened attention.

Through univariate analysis, we found significant differences in multiple indicators between the positive and negative groups of multidrug-resistant bacteria in the training cohort. We assessed the relevant indicators and discarded those with more than 20% missing values. Subsequently, we performed multivariate logistic regression, which included six indicators: secondary surgery, hospital stays, preoperative white blood cell (WBC) count, preoperative neutrophil count (N), postoperative WBC count, and postoperative C-reactive protein (CRP). Using these indicators, we constructed a diagnostic model that demonstrated good overall performance, with AUC values of 0.905 and 0.793 for the training and validation Cohort, respectively. The calibration curve indicated that the predicted values were in good agreement with the actual values. Decision curve analysis revealed that in both cohorts, the net benefit of treatment based on this model exceeded that of alternative strategies with threshold probabilities ranging from 0 to 1.

Reoperation (Secondary surgery) is a necessary intervention for managing severe complications that may arise after surgical procedures; however, it imposes a significant burden on patients. Multiple studies indicate that unexpected reoperations are predictive factors for prolonged hospital stays, increased complications, and reduced short-term survival rates (Xu et al., 2023; Luo et al., 2023; Zhuang et al., 2022). Patients with gastrointestinal fistulas usually experience unavoidable complications after surgery and may be forced to undergo a second surgical intervention shortly after the initial surgery. The reasons that led to the patient’s secondary surgery can be summarized in two points. On the one hand, the existing treatment of gastrointestinal fistulae is unsatisfactory, which may be due to poor initial abdominal drainage, leading to the possibility of severe intra-abdominal infection. On the other hand, salvage therapy is indispensable when patients experience life-threatening complications such as uncontrolled intra-abdominal hemorrhage. The need for a second surgery is a considerable setback for patients, often heralding adverse clinical outcomes, and consequently, it has become an independent risk factor for multidrug-resistant bacterial and fungal infections. Previous studies have found that the reoperation rate for patients with intestinal fistulas is approximately 15%, while the readmission rate ranges from 15% to 30%, which reflect the complexity of this condition and the challenges associated with its treatment (Christensen et al., 2021; Hatchimonji et al., 2020). Hospital stays for patients with gastrointestinal fistulas tend to be prolonged, often involving extended periods in the intensive care unit (ICU). This lengthy hospitalization undoubtedly provides a conducive environment for the establishment of multidrug-resistant bacteria, thereby serving as an independent risk factor for both multidrug-resistant bacterial and fungal infections, consistent with prior research (Shen et al., 2023). Preoperative white blood cell (WBC) counts, neutrophil counts (N), and postoperative WBC levels reflect the overall inflammatory status of the body. Previous studies have indicated a close relationship between these markers and the occurrence of multidrug-resistant bacterial infections (Yan et al., 2022). Both WBCs and neutrophils play critical roles in angiogenesis, hematopoiesis, wound healing, and inflammatory processes associated with infectious diseases (Carnevale et al., 2023). Procalcitonin (PCT) and C-reactive protein (CRP) are commonly used biological markers for inflammation and sepsis (Tian et al., 2023). Under normal physiological conditions, serum PCT concentrations are extremely low. PCT is synthesized in monocytes and the liver, and its production is stimulated by lipopolysaccharides and cytokines (Pournaghi et al., 2024). Conversely, CRP is produced by the liver in response to interleukin-6 (IL-6), which is generated during infection and inflammation. Studies show that in patients who develop postoperative infections, CRP levels increase rapidly. In patients responsive to antibiotic treatment, CRP levels typically decrease within 48 hours post-surgery. A CRP level exceeding 250 mg/L often indicates poorer clinical outcomes (Perrella et al., 2021). As significant indicators of inflammation, the combined use of CRP and PCT can effectively enhance the diagnostic efficiency for intra-abdominal infections (Song and Lu, 2021). In this study, univariate analysis demonstrated that both preoperative and postoperative PCT and CRP levels were significant; however, due to a certain proportion of missing data for preoperative PCT and CRP, these were not included in the multivariate analysis. The multivariate analysis revealed that postoperative CRP is an independent risk factor for predicting multidrug-resistant bacterial and fungal infections.

Our study has several limitations. First, as a retrospective study, it may be subject to some degree of selection bias. We obtained a limited number of case observations from a small sample size and a single center. Second, although our study was divided into a training cohort and a validation cohort, further prospective studies are necessary to validate the accuracy of our nomogram before widespread clinical application. Third, we did not include antibiotic use as a factor in our analysis due to the complexity of the information regarding antibiotic therapy. Many patients had already been on antibiotics for a period before transferring to our hospital, making statistical evaluation challenging. Consequently, we plan to conduct prospective randomized controlled studies for further exploration.

## 5 Conclusion

Our study indicate that in patients with gastrointestinal fistulas, perioperative infections are predominantly caused by multidrug-resistant Gram-negative bacteria, with carbapenemase-producing Enterobacteriaceae and multidrug-resistant Klebsiella pneumoniae emerging as particularly concerning issues. The independent risk factors for multidrug-resistant bacterial and fungal infections during the perioperative period in these patients include secondary surgery, prolonged hospital stays, preoperative white blood cell (WBC) counts, preoperative neutrophil counts, postoperative WBC counts, and postoperative C-reactive protein (CRP) levels. Developing a predictive model based on these indicators demonstrates good clinical applicability and high predictive accuracy, which can inform effective prevention and treatment strategies, facilitate rational antibiotic use, and improve patient outcomes.

## Data Availability

The original contributions presented in the study are included in the article/supplementary material, further inquiries can be directed to the corresponding author/s.
